# LSTM and GRU Neural Networks as Models of Dynamical Processes Used in Predictive Control: A Comparison of Models Developed for Two Chemical Reactors

**DOI:** 10.3390/s21165625

**Published:** 2021-08-20

**Authors:** Krzysztof Zarzycki, Maciej Ławryńczuk

**Affiliations:** Faculty of Electronics and Information Technology, Institute of Control and Computation Engineering, Warsaw University of Technology, Nowowiejska 15/19, 00-665 Warsaw, Poland; M.Lawrynczuk@ia.pw.edu.pl

**Keywords:** dynamical systems, LSTM and GRU neural networks, model predictive control

## Abstract

This work thoroughly compares the efficiency of Long Short-Term Memory Networks (LSTMs) and Gated Recurrent Unit (GRU) neural networks as models of the dynamical processes used in Model Predictive Control (MPC). Two simulated industrial processes were considered: a polymerisation reactor and a neutralisation (pH) process. First, MPC prediction equations for both types of models were derived. Next, the efficiency of the LSTM and GRU models was compared for a number of model configurations. The influence of the order of dynamics and the number of neurons on the model accuracy was analysed. Finally, the efficiency of the considered models when used in MPC was assessed. The influence of the model structure on different control quality indicators and the calculation time was discussed. It was found that the GRU network, although it had a lower number of parameters than the LSTM one, may be successfully used in MPC without any significant deterioration of control quality.

## 1. Introduction

In Model Predictive Control (MPC) [1,2], a dynamical model of the controlled process is used to predict its behaviour over a certain time horizon and to optimise the control policy. This problem formulation leads to very good control quality, much better than that in classical control methods. As a result, MPC methods have been used for a great variety of processes, e.g., chemical reactors [3], heating, ventilation and air conditioning systems [4], robotic manipulators [5], electromagnetic mills [6], servomotors [7], electromechanical systems [8] and stochastic systems [9]. It must be pointed out that satisfactory control is only possible if the model used is precise enough. Although there are numerous types of dynamical models, e.g., fuzzy systems, polynomials, and piecewise linear structures [10], neural networks of different kinds [11] are very popular due to their excellent accuracy and simple structure [12]. In particular, Recurrent Neural Networks (RNNs) [13,14,15,16] can serve as a model as they are able to give predictions over the required horizon.

In theory, RNNs can be extremely useful in various machine learning tasks in which the data are time-dependent such as modelling of time series, speech synthesis or video analysis. In contrast to the classical feedforward neural networks, RNNs can be used to create models and predictions from sequential data. However, in practice, their use is limited due to their one major drawback: the lack of long-term memory. RNNs have short-term memory capabilities; however, they tend to forget about the long-term input–output time dependencies during the backpropagation training. This problem is caused by the vanishing gradient phenomena, which was described in great detail in [17,18,19]. Many ways of limiting the vanishing gradient influence on the training process have been proposed, such as using different activation functions (such as ReLU) or branch normalisation. Another approach is to modify the network architecture in a way that improves the gradient flow during training. Residual Neural Networks (ResNets) proposed in [20] and the Long Short-Term Memory Network (LSTM) structure proposed first in [18] and its modification—the Gated Recurrent Unit (GRU) architecture proposed in [21]—can serve as examples.

The unique long-term memory properties of LSTM and GRU neural networks made them widely popular in a large variety of machine learning tasks. Example applications of the LSTM architecture are: data classification [22], speech recognition [23,24], handwriting recognition [25], speech synthesis [26], text coherence tests [27], biometric authentication and anomaly detection [28], detecting deception from gaze and speech [29] and anomaly detection [30]. Similarly, example applications of the GRU structure are: facial expression recognition [31], human activity recognition [32], cyberbullying detection [33], defect detection [34], human activity surveillance [35], automated classification of cognitive workload tasks [36] and speaker identification [37].

Recently, the LSTM networks have been also used to model dynamical processes. Examples are: a benchmark process [38], a pH reactor [39], a reverse osmosis plant [40], temperature control [41] or an autonomous mobility-on-demand system [42]. In all cited publications, it was shown that the LSTM models are able to approximate the properties of dynamical processes; the models have very good accuracy. Some of these models have been used for prediction in MPC [40,41,42]; very good control quality has been reported. Although GRU networks are similar to the LSTM ones and they have many successful applications in classification and detection tasks, as mentioned in the previous paragraph, they are very rarely used as models of dynamical processes, e.g., a tandem-wing quadplane drone model was discussed in [43]. Hence, two important questions should be formulated:

(a) What is the accuracy of the dynamical models based on the GRU networks, and how do they compare to the LSTM ones?
(b) How do the GRU dynamical models perform in MPC, and how do they compare to the LSTM-based MPC approach?

Both of these issues are worth considering since the GRU networks have a simpler architecture and a lower number of parameters than the LSTM ones.

This work has three objectives:

(a) A thorough comparison of LSTM and GRU neural networks as models of two dynamical processes, polymerisation and neutralisation (pH) reactors, is considered. An important question is whether or not the GRU network, although it has a simpler structure as the LSTM one, offers satisfying modelling accuracy;
(b) The derivation of MPC prediction equations for the LSTM and GRU models;
(c) The development of MPC algorithms for the two aforementioned processes with different LSTM and GRU models used for prediction. An important question is whether or not the GRU network offers control quality comparable to that possible when the more complex LSTM structure is applied.

Unfortunately, to the best of the authors’ knowledge, the efficiency of LSTM and GRU networks as dynamical models and their performance in MPC have not been thoroughly compared in the literature; typically, the LSTM structures are used [40,41,42].

The article is organised in the following way. Section 2 describes the structures of the LSTM and GRU neural networks. Section 3 defines the MPC optimisation task algorithm and details how the two discussed types of neural models are used for prediction in MPC. Section 4 thoroughly compares the efficiency of LSTM and GRU neural networks used as models of the two dynamical systems. Moreover, the efficiency of both considered model classes is validated in MPC. Finally, Section 5 summarises the whole article.

## 2. LSTM and GRU Neural Networks

### 2.1. The LSTM Neural Network

The LSTM approach aims to create a model that has a long-term memory and, at the same time, is able to forget about unimportant information in the training data. To achieve this, three main differences in comparison to classical RNNs are introduced:

- Two types of activation functions;
- A cell state that serves as the long-term memory of the neuron;
- The neuron is called a cell and has a complex structure consisting of four gates that regulate the information flow.

#### 2.1.1. Activation Functions

In the classical RRNs, the most commonly used activation function is the tanh type:

(1) $$\tanh\left(x\right)=\frac{e^{x}-e^{-x}}{e^{x}+e^{-x}}.$$

The output values of the hyperbolic tangent are in the range <−1,1>. This helps to regulate the data flow through the network and avoid the exploding gradient phenomena [17,44]. In the LSTM networks, the usage of tanh is kept; however, the sigmoid activation function is additionally implemented. This function is defined as:

(2) $$\sigma \left(x\right)=\frac{1}{1+e^{-x}}.$$

The output values of the sigmoid function are in the range of <0,1>. This allows the neural network to discard irrelevant information. If the output values are close to zero, they are not important and should be forgotten. If the values are close to one, they should be kept.

#### 2.1.2. Hidden State and Cell State

**Figure 1:**
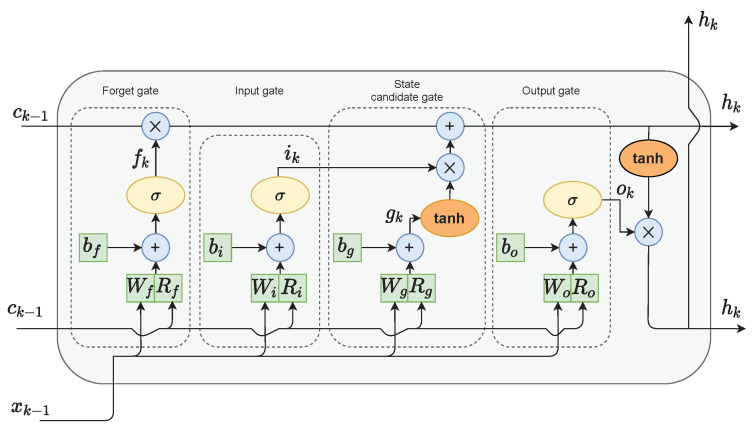
The LSTM cell structure.

In the classical RNN architecture, the hidden state is used as a memory of the network and an output of the hidden layer of the network. The LSTM networks additionally implement a cell state. In their case, the hidden state serves as a short-term working memory. On the other hand, the cell state is used as a long-term memory to keep information about important data from the past. As depicted in Figure 1, only a few linear operations are performed on the cell state. Therefore, the gradient flow during the backpropagation training is relatively undisturbed. This helps to limit the occurrence of the vanishing gradient problem.

#### 2.1.3. Gates

The LSTM network has the ability to modify the value of the cell state through a mechanism called gates. The LSTM cell shown in Figure 1 consists of four gates:

1. The forget gate *f* decides which values of the previous cell state should be discarded and which should be kept;
2. The input gate *i* selects values from the previous hidden state and the current input to update by passing them through the sigmoid function. The function product is then multiplied by the previous cell state;
3. The cell state candidate gate *g* first regulates the information flow in the network by using the tanh function on the previous hidden state and the current input. The product of tanh is multiplied by the input gate output to calculate the candidate for the current cell state. The candidate is then added to the previous cell state;
4. The output gate *o* first calculates the current hidden state by passing the previous hidden state and the current input through the sigmoid function to select which new information should be taken into account. Then, the current cell state value is passed through the tanh function. The products of both of those functions are finally multiplied.

#### 2.1.4. LSTM Layer Architecture

The LSTM layer of a neural network is composed of $n_{N}$ neurons. The layer has $n_{f}$ input signals. For a network used as a dynamical model of the process represented by the general equation:

(3) $$y\left(k\right)=f\left(x\left(k\right)\right)=f\left(u\left(k-1\right),\ldots ,u\left(k-n_{B}\right),y\left(k-1\right),\ldots ,y\left(k-n_{A}\right)\right)$$

this parameter can be written as $n_{f}=n_{A}+n_{B}$. The vector of the network’s input signals at the time instant *k* is then:

(4) $$x\left(k\right)=\left[\begin{array}{c} u(k-1)\\ \vdots \\ u(k-n_{B})\\ y(k-1)\\ \vdots \\ y(k-n_{A}) \end{array}\right].$$

When considering the entire LSTM network layer consisting of $n_{N}$ cells, the gates can be represented as vectors f, g, i, o, each of dimensionality $n_{N}\times 1$. The LSTM layer of the network contains also a number of weights. The symbol W denotes the weights associated with the input signals x; the symbol R denotes the so-called recursive weights, associated with the hidden state of the cell from the previous moment h(k−1); the symbol b denotes the constant (bias) components. The subscripts f, g, i or o appear next to all the weights; they indicate to which gate the weights belong. Network weights can be therefore written in matrix form as:

(5) $$W=\left[\begin{array}{c} W_{i}\\ W_{f}\\ W_{g}\\ W_{o} \end{array}\right],\;R=\left[\begin{array}{c} R_{i}\\ R_{f}\\ R_{g}\\ R_{o} \end{array}\right],\;b=\left[\begin{array}{c} b_{i}\\ b_{f}\\ b_{g}\\ b_{o} \end{array}\right].$$

The matrices $W_{i}$, $W_{f}$, $W_{g}$ and $W_{o}$ have dimensionality $n_{N}\times n_{f}$; the matrices $R_{i}$, $R_{f}$, $R_{g}$ and $R_{o}$ have dimensionality $n_{N}\times n_{N}$; the vectors $b_{i}$, $b_{f}$, $b_{g}$ and $b_{o}$ have dimensionality $n_{N}\times 1$.

At the time instant *k*, the following calculations are performed sequentially in the LSTM layer of the network:

(6) $$\begin{array}{cc} i(k) & =\sigma \left(W_{i}x\left(k\right)+R_{i}h\left(k-1\right)+b_{i}\right), \end{array}$$

(7) $$\begin{array}{cc} f(k) & =\sigma \left(W_{f}x\left(k\right)+R_{f}h\left(k-1\right)+b_{f}\right), \end{array}$$

(8) $$\begin{array}{cc} g(k) & =\tanh\left(W_{g}x\left(k\right)+R_{g}h\left(k-1\right)+b_{g}\right), \end{array}$$

(9) $$\begin{array}{cc} o(k) & =\sigma \left(W_{o}x\left(k\right)+R_{o}h\left(k-1\right)+b_{o}\right). \end{array}$$

The new cell state at the time instant *k* is then determined:

(10) $$\begin{array}{c} c(k)=f(k)\circ c(k-1)+i(k)\circ g(k). \end{array}$$

Finally, the hidden state at the time instant *k* can be calculated:

(11) $$\begin{array}{c} h(k)=o(k)\circ \tanh(c(k)). \end{array}$$

The symbol ∘ denotes the Hadamard product of the vectors. In other words, the vectors are multiplied elementwise. In Equation (10), this operation is used twice. The cell state from the previous time instant is multiplied by the values output by the forget gate. If those values are close to zero, the Hadamard product is close to zero as well, and therefore, the past information stored in the cell state is discarded. If the forget gate values are close to one, the past information becomes mostly unchanged. Then, the output of the input gate and cell candidate gate is pointwise multiplied. The purpose of this operation is similar. If the input gate values are close to zero, no new information is added to the cell state. Otherwise, the previous cell state values are updated with the values from the cell state candidate gate. In Equation (11), the Hadamard product is close to zero when the output gate values are close to zero. In this situation, the hidden state from the previous time instant becomes mostly unchanged. Otherwise, the new hidden state is updated with the new values from the cell state.

**Figure 2:**
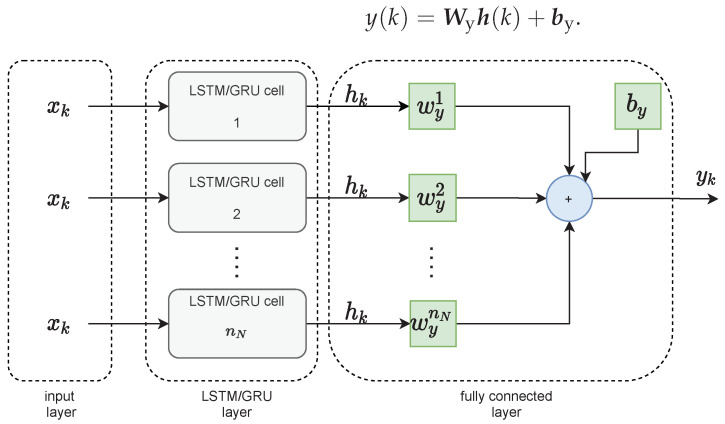
The topology of the LSTM and GRU networks.

The LSTM layer of the neural network is then connected to the fully connected layer, as shown in Figure 2. It has its weight vector $W_{y}$ of dimensionality $1\times n_{N}$ and bias $b_{y}$. The output of the network at the time instant *k* is calculated as follows:

(12) $$\begin{array}{c} y\left(k\right)=W_{y}h\left(k\right)+b_{y}. \end{array}$$

### 2.2. The GRU Neural Network

The GRU network is a modification of the LSTM concept, which aims to reduce to network’s computational cost. There are some differences between the architectures, mainly:

1. The GRU cell lacks the output gate; therefore, it has fewer parameters;
2. The usage of the cell state is discarded. The hidden state serves both as the working and long-term memory of the network.

**Figure 3:**
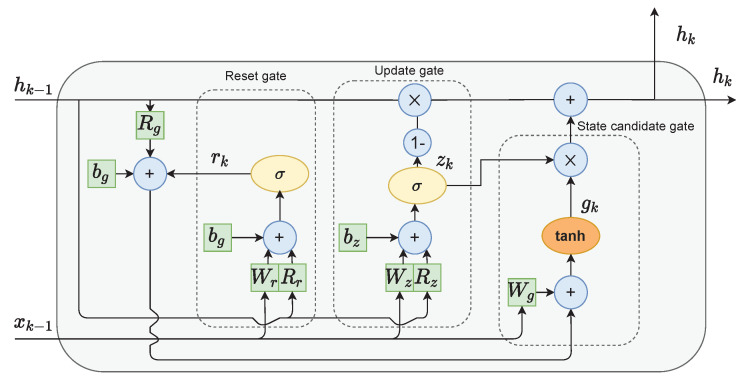
The GRU cell structure.

The single-GRU cell layout is presented in Figure 3. It consists of three gates:

1. The reset gate *r* is used to select which information to discard from the previous hidden state and input values;
2. The role of the update gate *z* is to select which information from the previous hidden state should be kept and passed along to the next steps;
3. Candidate state gate *g* calculates the candidate for the future hidden state. This is done by firstly multiplying the previous state with the reset gate’s output. This step can be interpreted as forgetting unimportant information from the past. Next, new data form the input are added to the remaining information. Finally, the tanh function is applied to the data to regulate the information flow.

The current hidden state $h_{k}$ is calculated as follows. Firstly, the output from the update gate *z* is subtracted from one and then multiplied with the previous state $h_{k-1}$. Then, the state candidate g is multiplied by the unchanged output from the update gate *z*. The results of both of those operations are finally added. This means that if the values output from update gate *z* are close to zero, more new information is added to the current state *h*. Alternatively, if the values output from update gate *z* are close to one, the current state is mostly kept as it was in the previous time iterations.

When considering the whole GRU layer of $n_{N}$ cells, the weight matrices $W_{r}$, $W_{z}$, $W_{o}$ have dimensions $n_{N}\times n_{f}$, matrices $R_{r}$, $R_{z}$, $R_{g}$ have dimensions $n_{N}\times n_{N}$, and vectors $b_{r}$, $b_{z}$, $b_{g}$ have dimensions $n_{N}\times 1$. The matrices can be written as:

(13) $$W=\left[\begin{array}{c} W_{r}\\ W_{z}\\ W_{g} \end{array}\right],\;R=\left[\begin{array}{c} R_{r}\\ R_{z}\\ b_{g} \end{array}\right],\;b=\left[\begin{array}{c} b_{r}\\ b_{z}\\ b_{g} \end{array}\right].$$

The following calculations are performed at the sampling time *k*:

(14) $$\begin{array}{cc} r(k) & =\sigma \left(W_{r}x\left(k\right)+R_{r}h\left(k-1\right)+b_{r}\right), \end{array}$$

(15) $$\begin{array}{cc} z(k) & =\sigma \left(W_{z}x\left(k\right)+R_{z}h\left(k-1\right)+b_{z}\right), \end{array}$$

(16) $$\begin{array}{cc} g(k) & =\tanh\left(W_{g}x\left(k\right)+r\left(k\right)\circ \left(R_{g}h\left(k-1\right)\right)+b_{g}\right), \end{array}$$

(17) $$\begin{array}{cc} h(k) & =\left(1_{n_{N}\times 1}-z\left(k\right)\right)\circ g\left(k\right)+z\left(k\right)\circ h\left(k-1\right). \end{array}$$

Similar to the LSTM layer, the GRU layer of the neural network is then connected to the fully connected layer. It has its weight vector $W_{y}$ of dimensionality $1\times n_{N}$ and a constant component $b_{y}$. The output of the network at the time *k* is determined by the hidden state of all cells of the GRU layer multiplied by the weights of the fully connected layer, respectively, according to the following relation:

(18) $$\begin{array}{c} y\left(k\right)=W_{y}h\left(k\right)+b_{y}. \end{array}$$

## 3. LSTM and GRU Neural Networks in Model Predictive Control

The manipulated variable, i.e., the input of the controlled process, is denoted by *u*, while the controlled one, i.e., the process output, is denoted by *y*. A good control algorithm is expected to calculate the value of the manipulated variable, which leads to fast control, i.e., the process output should follow the changes of the set-point. Moreover, since fast control usually requires abrupt changes of the manipulated variables, which may be dangerous for the actuator, such situations should be penalised. Finally, it is necessary to take some constraints; they are usually imposed on the magnitude and the rate of change of the manipulated variable. In some cases, constraints can also be imposed on the process output variable.

### 3.1. The MPC Problem

The vector of decision variables calculated online at each sampling instant of MPC is defined as the increments of the manipulated variable:

(19) $$▵u\left(k\right)=\left[\begin{array}{c} ▵u(k|k)\\ \vdots \\ ▵u(k+N_{u}-1|k) \end{array}\right]$$

where the control horizon is denoted by $N_{u}$. The general MPC optimisation problem is:

(20) $$\begin{array}{cc} \; & \min_{▵u(k)}\left\{J\left(k\right)=\sum_{p=1}^{N}\left(y^{\mathrm{sp}}\left(k+p|k\right)-\hat{y}\left(k+p|k\right)\right)+\lambda \sum_{p=0}^{N_{u}-1}{\left(▵u(k+p|k)\right)}^{2}\right\}\\ \; & \mathrm{subject}\;\mathrm{to}\\ \; & u^{\min}\leq u\left(k+p|k\right)\leq u^{\max},\;p=0,\ldots ,N_{u}-1\\ \; & ▵u^{\min}\leq ▵u\left(k+p|k\right)\leq ▵u^{\max},\;p=0,\ldots ,N_{u}-1\\ \; & y^{\min}\leq \hat{y}\left(k+p|k\right)\leq y^{\max},\;p=1,\ldots ,N. \end{array}$$

The cost function can be divided into two parts. The first part describes the control error, which is defined as the sum of the differences between the set-point value $y^{\mathrm{sp}}\left(k+p|k\right)$ and the output prediction $\hat{y}\left(k+p|k\right)$ over the prediction horizon *N*. The (k+p|k) notation should be interpreted as follows: the prediction of the moment in the future k+p is calculated in the current moment *k*. The second part of the cost function consists of the change of the manipulated variables multiplied by the weighting coefficient λ. When the whole cost function is taken into account, one can observe that it minimises both control errors and the change of control signals. Weighting coefficient λ is used to fine-tune the procedure.

The constraints of the MPC optimisation problem are as follows:

- The magnitude constraints $u^{\min}$ and $u^{\max}$ are enforced on the manipulated variable over the control horizon $N_{u}$;
- The constraints $▵u^{\min}$ and $▵u^{\max}$ are imposed on the increments of the same variable over the control horizon $N_{u}$;
- The constraints put on the predicted output variable $y^{\min}$ and $y^{\max}$ over the prediction horizon *N*.

When the optimisation procedure calculates the decision vector (Equation (19)) from Equation (20), the first element of it is applied to the process. The most common way of this application is given by the following equation:

(21) $$\begin{array}{c} u(k)=\Delta u(k|k)+u(k-1|k). \end{array}$$

The whole computational scheme is then repeated at the next sampling instants.

In MPC [2], the general prediction equation for the sampling instant k+p is:

(22) $$\hat{y}\left(k+p|k\right)=y\left(k+p|k\right)+d\left(k\right)$$

where p=1,…,N. The output of the model for the sampling instant k+p calculated at the current instant *k* is y(k+p|k), and the current estimation of the unmeasured disturbance acting on the process output is d(k). Typically, it is assumed that the disturbance is constant over the whole prediction horizon, and its value is determined as the difference between the real (measured) value of the process output and the model output calculated using the process input and output signals up to the sampling instant k−1:

(23) $$d\left(k\right)=y_{m}\left(k\right)-y\left(k|k-1\right).$$

### 3.2. The LSTM Neural Network in MPC

In the case of the LSTM model, to determine the predicted output, it is necessary to first calculate the prediction values of the cell state given by Equations (6)–(10) in the following way:

(24) $$\begin{array}{cc} \hat{c}\left(k+1|k\right)= & \sigma \left(W_{f}x\left(k+1|k\right)+R_{f}h\left(k\right)+b_{f}\right)\circ c\left(k\right)\\ & +\sigma \left(W_{i}x\left(k+1|k\right)+R_{i}h\left(k\right)+b_{i}\right)\\ & \times \tanh\left(W_{g}x\left(k+1|k\right)+R_{g}h\left(k\right)+b_{g}\right) \end{array}$$

(25) $$\begin{array}{cc} \hat{c}\left(k+2|k\right) & =\sigma \left(W_{f}x\left(k+2|k\right)+R_{f}\hat{h}\left(k+1|k\right)+b_{f}\right)\circ \hat{c}\left(k+1|k\right)\\ \; & \;+\sigma \left(W_{i}x\left(k+2|k\right)+R_{i}\hat{h}\left(k+1|k\right)+b_{i}\right)\\ \; & \;\times \tanh\left(W_{g}x\left(k+2|k\right)+R_{g}\hat{h}\left(k+1|k\right)+b_{g}\right) \end{array}$$

$$\begin{array}{c} \vdots \end{array}$$

(26) $$\begin{array}{cc} \hat{c}\left(k+p|k\right) & =\sigma \left(W_{f}x\left(k+p|k\right)+R_{f}\hat{h}\left(k+p-1|k\right)+b_{f}\right)\circ \hat{c}\left(k+p-1|k\right)\\ \; & \;+\sigma \left(W_{i}x\left(k+1|k\right)+R_{i}\hat{h}\left(k+p-1|k\right)+b_{i}\right)\\ \; & \;\times \tanh\left(W_{g}x\left(k+p|k\right)+R_{g}\hat{h}\left(k+p-1|k\right)+b_{g}\right). \end{array}$$

Using Equations (6)–(9) and Equation (11), one can then calculate the prediction of the hidden state:

(27) $$\begin{array}{cc} \hat{h}\left(k+1|k\right) & =\sigma \left(W_{o}x\left(k+1|k\right)+R_{o}h\left(k\right)\circ \tanh(\hat{c}\left(k+1|k\right)\right) \end{array}$$

(28) $$\begin{array}{cc} \hat{h}\left(k+2|k\right) & =\sigma \left(W_{o}x\left(k+2|k\right)+R_{o}\hat{h}\left(k+1|k\right)\circ \tanh(\hat{c}\left(k+2|k\right)\right) \end{array}$$

$$\begin{array}{c} \vdots \end{array}$$

(29) $$\begin{array}{cc} \hat{h}\left(k+p|k\right) & =\sigma \left(W_{o}x\left(k+p|k\right)+R_{o}\hat{h}\left(k+p-1|k\right)\circ \tanh(\hat{c}\left(k+p|k\right)\right). \end{array}$$

Finally, the prediction of the output signal can be calculated based on Equations (18) and (32) as:

(30) $$\begin{array}{cc} y(k+1|k) & =W_{y}\hat{h}\left(k+1|k\right)+b_{y}+d\left(k\right) \end{array}$$

(31) $$\begin{array}{cc} y(k+2|k) & =W_{y}\hat{h}\left(k+2|k\right)+b_{y}+d\left(k\right) \end{array}$$

$$\begin{array}{c} \vdots \end{array}$$

(32) $$\begin{array}{cc} y(k+p|k) & =W_{y}\hat{h}\left(k+p|k\right)+b_{y}+d\left(k\right). \end{array}$$

Taking into account the input vector of the network (Equation (4)), for prediction over the prediction horizon, the vector of arguments of the network is:

(33) $$\begin{array}{cc} x(k+1|k) & ={\left[u\left(k|k\right)\;u\left(k-1\right)\;\cdots \;u\left(k-n_{B}+1\right)\;y\left(k\right)\;y\left(k-1\right)\;\cdots \;y\left(k-n_{A}+1\right)\right]}^{T} \end{array}$$

(34) $$\begin{array}{cc} x(k+2|k) & ={\left[u\left(k+1|k\right)\;u\left(k|k\right)\;\cdots \;u\left(k-n_{B}+2\right)\;\hat{y}\left(k+1|k\right)\;y\left(k\right)\;\cdots \;y\left(k-n_{A}+2\right)\right]}^{T} \end{array}$$

$$\begin{array}{c} \vdots \end{array}$$

(35) $$\begin{array}{ccc} x(k+p|k)= & [u\left(k+p-1|k\right)\;u\left(k+p-2|k\right)\;\cdots u\left(k-n_{B}+p\right) & \\ \; & \hat{y}\left(k+p-1|k\right)\;\hat{y}\left(k+p-2|k\right))\;\cdots \;y\left(k-n_{A}+p-1\right){]}^{T}. \end{array}$$

### 3.3. The GRU Neural Network in MPC

There is no cell state in the GRU neural networks, and therefore, to calculate the predicted output signal values $\hat{y}$, only the prediction of hidden state *h* is necessary to evaluate first. This is performed based on Equations (14)–(17) in the following way:

(36) $$\begin{array}{cc} \hat{h}\left(k+1|k\right) & =\left[1_{nN\times 1}-\sigma \left(W_{z}x\left(k+1|k\right)+R_{z}h\left(k\right)+b_{z}\right)\right]\\ \; & \;\circ \tanh\left[W_{g}x\left(k+1|k\right)+\sigma \left(W_{r}x\left(k+1|k\right)+R_{r}h\left(k\right)+b_{r}\right)\circ \left(R_{g}h\left(k\right)\right)+b_{g}\right]\\ \; & \;+\sigma \left(W_{z}x\left(k+1|k\right)+R_{z}h\left(k\right)+b_{z}\right)\circ h\left(k\right)) \end{array}$$

(37) $$\begin{array}{cc} \hat{h}\left(k+2|k\right) & =\left[1_{nN\times 1}-\sigma \left(W_{z}x\left(k+2|k\right)+R_{z}\hat{h}\left(k+1|k\right)+b_{z}\right)\right]\\ \; & \;\circ \tanh[W_{g}x\left(k+2|k\right)+\sigma \left(W_{r}x\left(k+2|k\right)+R_{r}\hat{h}\left(k+1|k\right)+b_{r}\right)\\ \; & \;\circ \left(R_{g}\hat{h}\left(k+1|k\right)\right)+b_{g}]\\ \; & \;+\sigma \left(W_{z}x\left(k+2|k\right)+R_{z}\hat{h}\left(k+1|k\right)+b_{z}\right)\circ \hat{h}\left(k+1|k\right)) \end{array}$$

$$\begin{array}{c} \vdots \end{array}$$

(38) $$\begin{array}{cc} \hat{h}\left(k+p|k\right) & =\left[1_{nN\times 1}-\sigma \left(W_{z}x\left(k+p|k\right)+R_{z}\hat{h}\left(k+p-1|k\right)+b_{z}\right)\right]\\ \; & \;\circ \tanh[W_{g}x\left(k+p|k\right)+\sigma \left(W_{r}x\left(k+p|k\right)+R_{r}\hat{h}\left(k+p-1|k\right)+b_{r}\right)\\ \; & \;\circ \left(R_{g}\hat{h}\left(k+p-1|k\right)\right)+b_{g}]\\ \; & \;+\sigma \left(W_{z}x\left(k+p|k\right)+R_{z}\hat{h}\left(k+p-1|k\right)+b_{z}\right)\circ \hat{h}\left(k+p-1|k\right)) \end{array}$$

where $1_{n_{N}\times 1}$ is an identity matrix with dimensions $n_{N}\times 1$. The prediction of the output signal Equation (32), as well as the input vector Equation (35) are the same as in the LSTM neural network model.

The proposed MPC control procedure may be summarised as follows:

1. The estimated disturbance d(k) is calculated from Equation (22):
  a. In the case of the LSTM network, the model output y(k|k−1) is calculated from Equations (6)–(12);
  b. In the case of the GRU network, the model output is calculated from Equations (14)–(17) and Equation (12);
2. The MPC optimisation task is then performed. To calculate the output prediction, the cell and hidden state prediction must be calculated first:
  a. For the LSTM model, the predictions are calculated from Equations (24)–(32);
  b. For the GRU, the model state prediction are calculated from Equations (36)–(38) and the output prediction is generated as shown in Equation (32). The cost function is the same for both models and is given by Equation (20);
3. The first element of the calculated decision vector (Equation (19)) is applied to the process, i.e., u(k)=▵u(k|k)+u(k−1|k).

## 4. Results of the Simulations

In order to compare the accuracy of the LSTM and GRU networks and their efficiency in MPC, we considered two dynamical systems: a polymerisation reactor and a neutralisation (pH) reactor.

### 4.1. Description of the Dynamical Systems

First, two considered processes are briefly described. Moreover, a short description of the data preparation procedure is given.

#### 4.1.1. Benchmark 1: The Polymerisation Reactor

The first considered benchmark was a polymerisation reaction taking place in a jacketed continuous stirred-tank reactor. The reaction was the free-radical polymerisation of methyl methacrylate with azo-bis-isobutyronitrile as the initiator and toluene as the solvent. The process input was the inlet initiator flow rate $F_{I}$ (m${}^{3}$ h${}^{-1}$); the output was the value of Number Average Molecular Weight (NAMW) of the product(kg kmol${}^{-1}$). The detailed fundamental model of the process was given in [45]. The process was nonlinear: in particular, its static gain depended on the operating point. The polymerisation reactor is frequently used to evaluate model identification algorithms and advanced nonlinear control methods, e.g., [12,45,46].

The fundamental model of the polymerisation process, comprising four nonlinear differential equations, was solved using the Runge–Kutta 45 method to obtain training and validation and test datasets, each of them having 2000 samples. After each 50 samples, there was a step change of the control signal. The magnitude of the control signal was chosen randomly. Next, since process input and output signals had different magnitudes, these signals were scaled in the following way:

(39) $$u=100\left(F_{I}-{\bar{F}}_{I}\right),\;y=0.0001\left(\mathrm{NAMW}-\bar{\mathrm{NAMW}}\right)$$

where ${\bar{F}}_{I}=0.016783$ and $\bar{\mathrm{NAMW}}=$ 20,000 denote the values of the variables at the nominal operating point. The sampling time was 1.8 s.

#### 4.1.2. Benchmark 2: The Neutralisation Reactor

The second considered benchmark was a neutralisation reactor. The process input was the base (NaOH) streamflow-rate $q_{1}$ (mL/s); the output was the value of the pH of the product. The detailed fundamental model of the process was given in [47]. The process was nonlinear since its static and dynamic properties depended on the operating point. Hence, it is frequently used as a good benchmark to evaluate model identification algorithms and advanced nonlinear control methods, e.g., [46,47,48].

The fundamental model of the neutralisation process, comprising two nonlinear differential equations and a nonlinear algebraic equation, was solved using the Runge–Kutta 45 method to obtain training and validation and test datasets, each of them having 2000 samples. After each 50 samples, there was a step change of the control signal. The magnitude of the control signal was chosen randomly. The process signals were scaled in the following way:

(40) $$u=q_{1}-{\bar{q}}_{1},\;y=\mathrm{pH}-\bar{\mathrm{pH}}$$

where ${\bar{q}}_{1}=15.5$ and $\bar{\mathrm{pH}}=7$ denote the values of the variables at the nominal operating point. The sampling time was 10 s.

### 4.2. LSTM and GRU Neural Networks for Modelling of Polymerisation and Neutralisation Reactors

A number of LSTM and GRU models were trained for the two considered dynamic processes. All models were trained using the Adam optimisation algorithm. The maximum number of training epochs (iterations) was:

- 500 for the models with $n_{N}\leq 3$;
- 750 for the models with $3<n_{N}\leq 7$;
- 1000 for the models with $7<n_{N}$.

The training procedure was performed as follows:

1. The order of the dynamics of the LSTM model was set to $n_{A}=n_{B}=1$. The number of neurons in the hidden layer was set to $n_{N}=1$. For the considered configuration, ten models were trained, and the best one was chosen;
2. The number of neurons was increased to two. Ten models were trained, and the best was chosen. This procedure was repeated until the number of neurons reached $n_{N}=30$;
3. The first two steps were repeated with the increased order of the dynamics $n_{A}=n_{B}=2$, $n_{A}=n_{B}=3$.

It is important to stress that setting the order of the dynamics to higher than $n_{A}=n_{B}=3$ did not result in any significant increase of the modelling quality. Therefore, further experiments with $n_{A}=n_{B}>3$ are not presented.

It is an interesting question if LSTM and GRU models without recurrent input signals $y\left(k-1\right)\cdots y\left(k-n_{A}\right)$ can perform well in modelling tasks. In theory, the recurrent nature of hidden state *h* should be sufficient to ensure good model quality. To verify this expectation, an additional series of models was trained. The training procedure was similar to the one described above, the only difference being that now, the model order of the dynamics was first set to $n_{A}=0$, $n_{B}=1$, then increased to $n_{A}=0$, $n_{B}=2$ and, finally, to $n_{A}=0$, $n_{B}=3$.

The quality of all trained models was then validated with the mean squared error chosen as the quality index. The models were validated in the nonrecurrent Autoregressive with eXogenous input (ARX) mode and the Output Error (OE) recurrent mode. The model input vectors for the two considered cases are:

(41) $$\begin{array}{c} x_{\mathrm{ARX}}\left(k\right)=\left[\begin{array}{c} u(k-1)\\ \vdots \\ u(k-n_{B})\\ y_{\mathrm{data}}\left(k-1\right)\\ \vdots \\ y_{\mathrm{data}}\left(k-n_{A}\right) \end{array}\right]\;\;\;x_{\mathrm{rec}}\left(k\right)=\left[\begin{array}{c} u(k-1)\\ \vdots \\ u(k-n_{B})\\ y_{\mathrm{model}}\left(k-1\right)\\ \vdots \\ y_{\mathrm{model}}\left(k-n_{A}\right) \end{array}\right]. \end{array}$$

It is important to stress that in the case of the models with $n_{A}=0$, the ARX and OE modes were the same.

**Figure 4:**
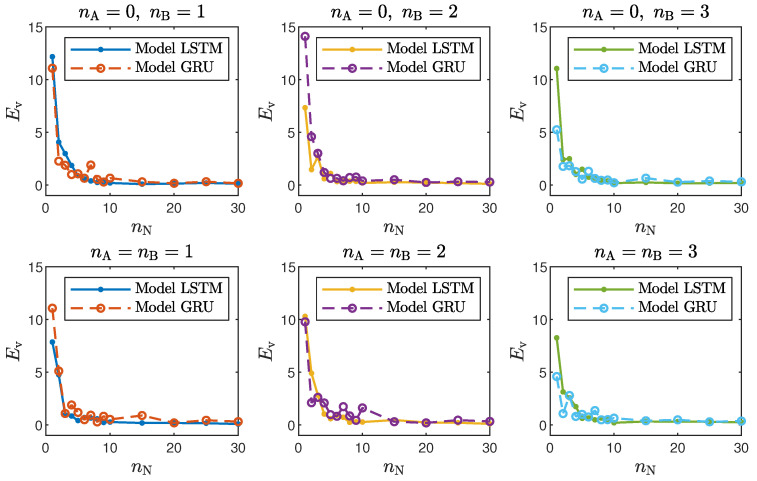
The polymerisation reactor: LSTM and GRU model validation errors for different numbers of neurons $n_{N}$.

**Figure 5:**
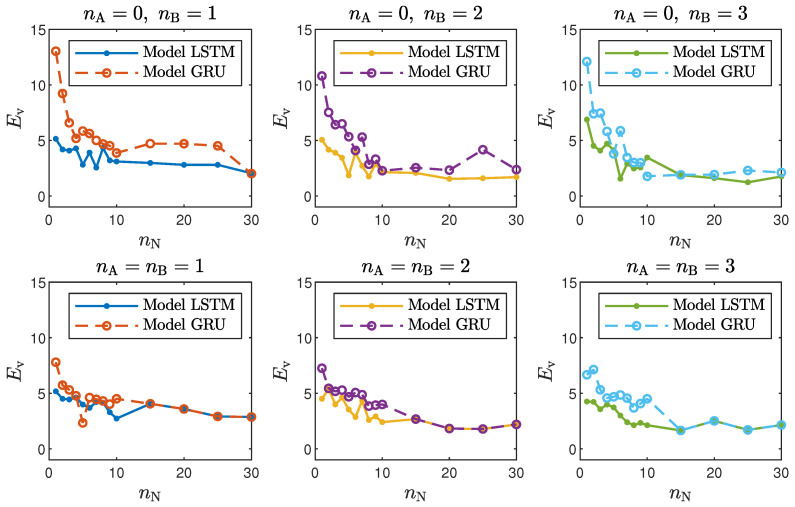
The neutralisation reactor: LSTM and GRU model validation errors for different numbers of neurons $n_{N}$.

Taking into account the objective of this work, it is interesting to compare the accuracy of the LSTM and GRU models with different structures, defined by the number of neurons, $n_{N}$, and the order of the model dynamics, determined by $n_{A}$ and $n_{B}$. For the polymerisation reactor, the results for the chosen networks are given in Table 1 and Table 2, and Figure 4 depicts the model validation errors for all considered numbers of neurons. For the neutralisation reactor, the results for the chosen networks are given in Table 3 and Table 4, and Figure 5 depicts the model validation errors for all considered numbers of neurons. The following notation is used:

- $E_{t}$ is the the mean squared error for the training dataset in ARX mode;
- $E_{v}$ is the the mean squared error for the validation dataset in ARX mode;
- $E_{t}^{\mathrm{rec}}$ is the the mean squared error for the training dataset in recurrent mode;
- $E_{v}^{\mathrm{rec}}$ is the the mean squared error for the validation dataset in recurrent mode.

**Figure 6:**
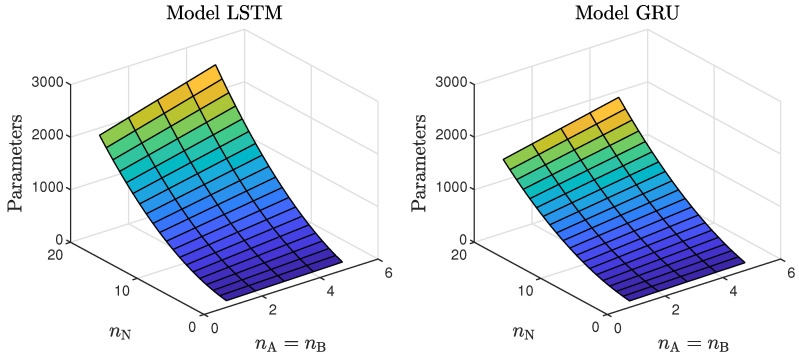
The number of the parameters of the LSTM and GRU models as a function of the number of neurons and the order of the dynamics determined by $n_{A}=n_{B}$.

**Figure 7:**
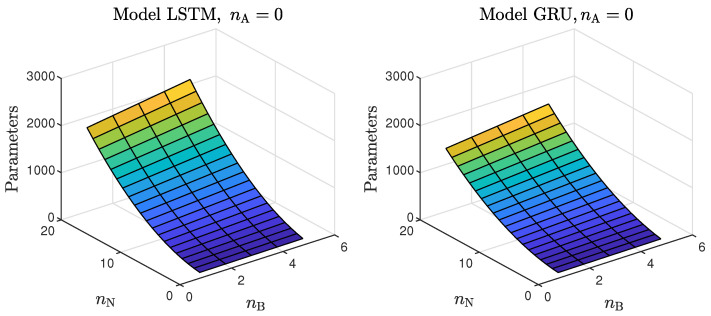
The number of parameters of the LSTM and GRU models as a function of the number of neurons and the order of the dynamics determined by $n_{B}$; $n_{A}=0$.

**Figure 8:**
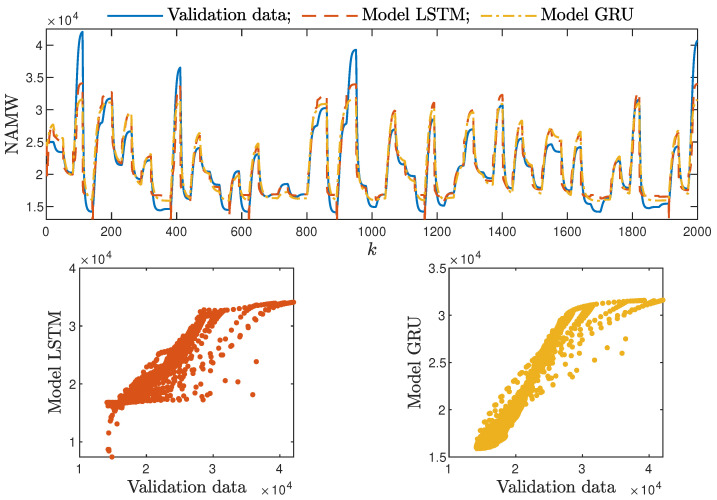
The polymerisation reactor: the validation dataset vs. the output of the LSTM and GRU models for $n_{N}=1$, $n_{A}=n_{B}=1$.

**Figure 9:**
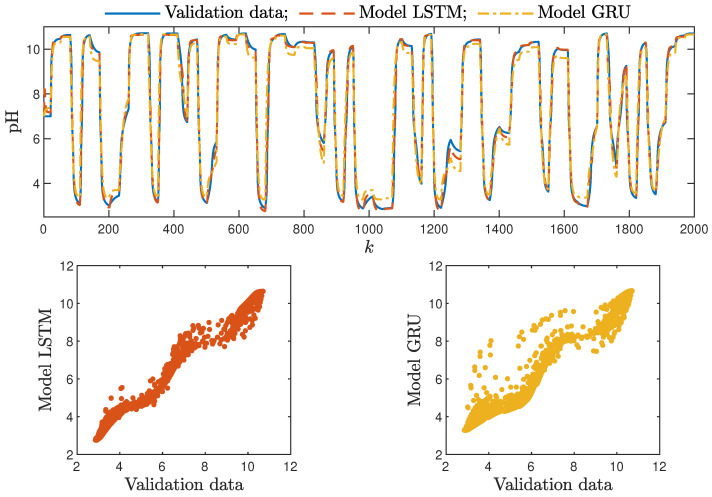
The neutralisation reactor: the validation dataset vs. the output of the LSTM and GRU models for $n_{N}=1$, $n_{A}=n_{B}=1$.

**Figure 10:**
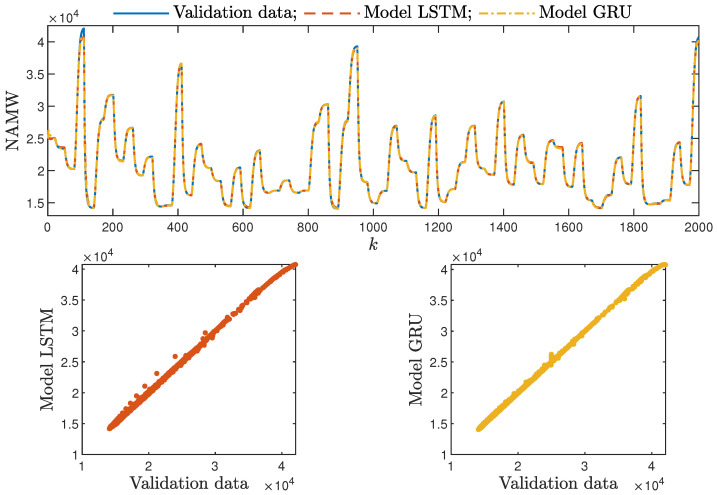
The polymerisation reactor: the validation dataset vs. the output of the LSTM and GRU models for $n_{N}=9$, $n_{A}=0$, $n_{B}=1$.

**Figure 11:**
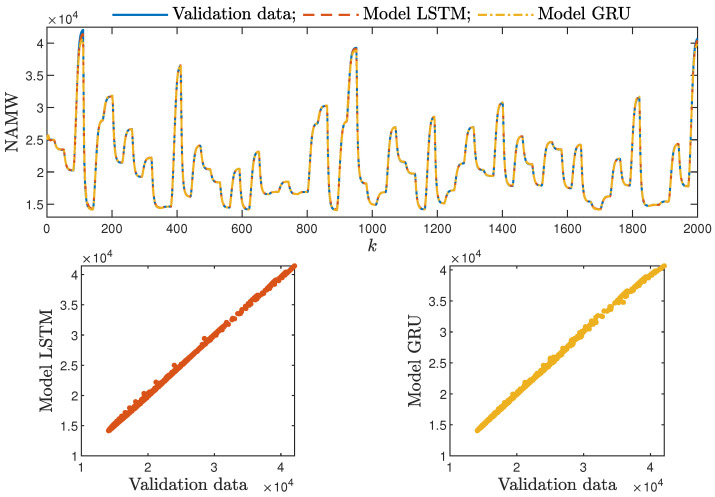
The polymerisation reactor: the validation dataset vs. the output of the LSTM and GRU models for $n_{N}=10$, $n_{A}=n_{B}=1$.

**Figure 12:**
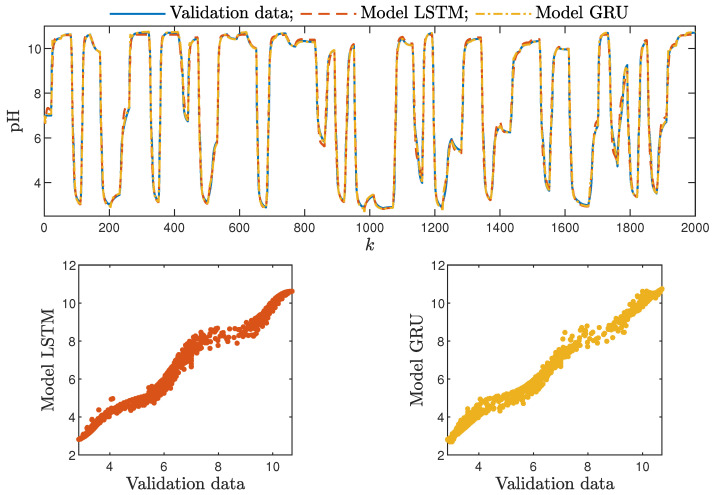
The neutralisation reactor: the validation dataset vs. the output of the LSTM and GRU models for $n_{N}=5$, $n_{A}=n_{B}=1$.

**Figure 13:**
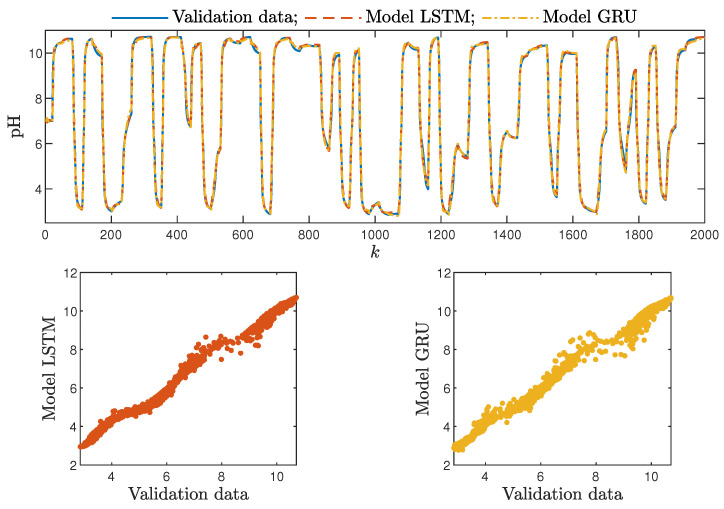
The neutralisation reactor: the validation dataset vs. the output of the LSTM and GRU models for $n_{N}=8$, $n_{A}=0$, $n_{B}=3$.

The presented results can be summarised in the following way:

- In the case of the polymerisation reactor, the results achieved with the LSTM and GRU networks were comparable. As seen in Figure 4, the means squared errors were similar for every combination of $n_{A}$, $n_{B}$ and $n_{N}$;
- In the case of the neutralisation reactor, the LSTM models ensured a better quality of modelling, especially for models with a low number of parameters. However, as seen in Figure 5, as the number of neurons increased, this difference became more and more negligible. This is again not surprising. GRU networks have less parameters than LSTM networks. Therefore, GRU models with a low number of neurons and a low order of the dynamics performed worse than their LSTM counterparts. As the models became bigger and more complex, the difference between their quality decreased.
- Models with a higher numbers of neurons (15–30) ensured the best and most consistent modelling quality. This is not surprising, as the number of model parameters is directly proportional to the capacity to reproduce the behaviour of more complex processes. However, this can also be a main drawback of complex models, because of the enormous number of parameters, as shown in Figure 6 and Figure 7, increases their computational cost significantly;
  These models had too few parameters to accurately represent the behaviour of the processes under study;
- For the models with a medium number of neurons (3–10), the modelling quality was not consistent. In some cases, it was quite poor; in others, it even outperformed models with a huge number of neurons (an example can be found in Table 4, the GRU network with $n_{A}=n_{B}=1$$n_{N}=5$). One can conclude that this group of models has a structure complex enough to represent the behaviour of the systems under investigation. The training procedure must be, however, performed many times, as training may sometimes not be successful. In other words, if the goal is to find the model with the minimum number of parameters and good quality, the medium-sized models are the best option;
- Models with a low (1–2) number of neurons did not ensure a good modelling quality regardless of the neural network type and the model order of the dynamics, as shown in Figure 8 and Figure 9.
- Interestingly enough, the order of the dynamics of the model seemed not to greatly impact the modelling quality. Models with higher order were most commonly only slightly better than those with $n_{A}=n_{B}=1$. Only in the case of the neutralisation reactor with $n_{A}=0$ in Table 3 could a noticeable improvement be observed when $n_{B}$ was set to two. The unique long-term memory quality of the networks under study may be a cause of this phenomenon. The information about the important previous input and output signals from the past can be kept inside the hidden and cell states, and therefore, the networks can perform very well with only the most recent input values (i.e., $n_{A}=0$, $n_{B}=1$);

Based on the observations summarised above, it can be concluded that it is a good practice to train a model with a medium number of neurons and a low order of the dynamics. This approach may require many training trials, but as a result, the model has a relatively low number of parameters; therefore, a lower computational cost can be achieved. A direct comparison of the polymerisation reactor models can be seen in Figure 10 and Figure 11. Both models performed very well, and the modelling errors were minimal. A similar comparison for the pH reactor can be seen in Figure 12 and Figure 13. The modelling quality was again very satisfactory. Here, it is important to stress that in the case of the GRU model with $n_{A}=0$, it was necessary to choose one with a higher order of the dynamics to achieve results similar to those ensured by the simpler LSTM models.

### 4.3. LSTM and GRU Neural Network for the MPC of Polymerisation and Neutralisation Reactors

A few of the best-performing models were been chosen with the aim of being applied in the MPC control scheme for prediction. First, let us describe the tuning procedure of the MPC controller. It starts with the selection of the prediction horizon. It should be long enough to cover the dynamic behaviour of the process. However, if the horizons are too long, the computation cost of the optimisation task increases. The control horizon cannot be too short since it gives insufficient control quality, while its lengthening also increases the computational burden. The process of tuning was therefore as follows:

- The constant weighting coefficient λ=1 was assumed;
- The prediction horizon *N* and the control horizon $N_{u}$ were set to have the same, arbitrarily chosen lengths. If the controller was not working properly, both horizons were lengthened;
- The prediction horizon was gradually shortened, and its minimal possible length was chosen (with the condition $N_{u}=N$);
- The effect of changing the length of the control horizon on the resulting control quality was then assessed experimentally (e.g., assuming successively $N_{u}=1,2,3,4,5,10,\ldots ,N$). The shortest possible control horizon was chosen;
- Finally, after determining the horizon’s lengths, the weighting coefficient λ was adjusted.

After applying the tuning procedure on both processes under study, the following settings were determined:

- N=10, $N_{u}=5$, λ=0.5 for the polymerisation process;
- N=10, $N_{u}=3$, λ=0.5 for the neutralisation process.

Simulations of the MPC algorithms were performed with MATLAB. For optimisation, the fmincon() function was used with the following settings:

- Optimisation algorithm—Sequential Quadratic Programming (SQP);
- Finite differences type—centred.

**Figure 14:**
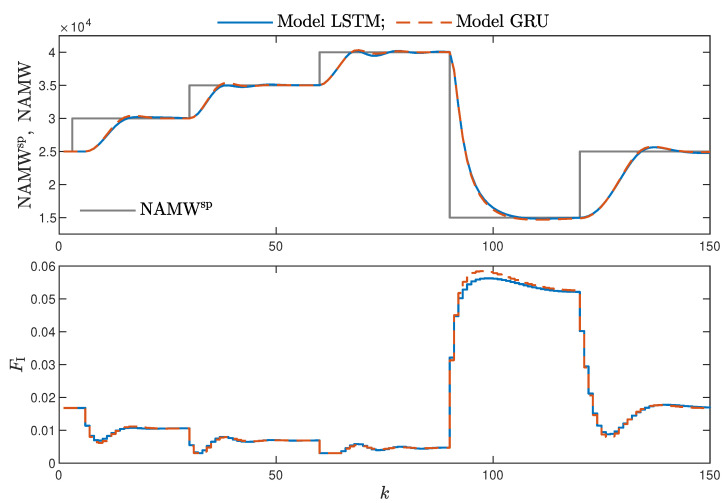
The polymerisation reactor: MPC results with the LSTM and GRU models $n_{N}=9$, $n_{A}=0$, $n_{B}=1$.

**Figure 15:**
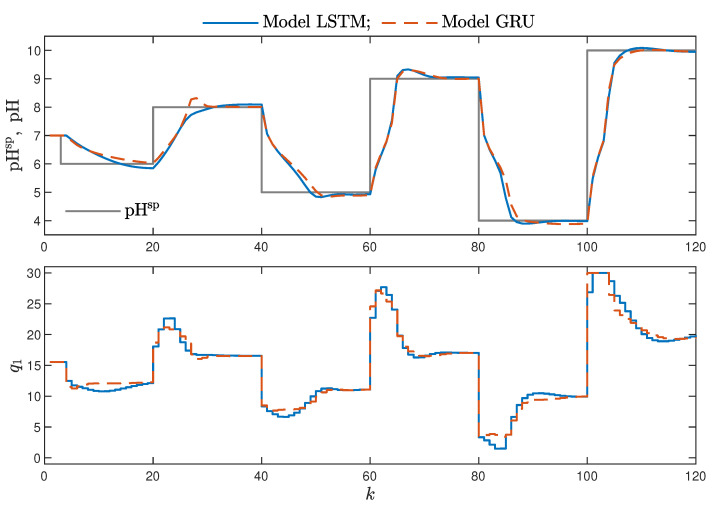
The neutralisation reactor: MPC results with the LSTM and GRU models for $n_{N}=8$, $n_{A}=0$, $n_{B}=3$.

**Figure 16:**
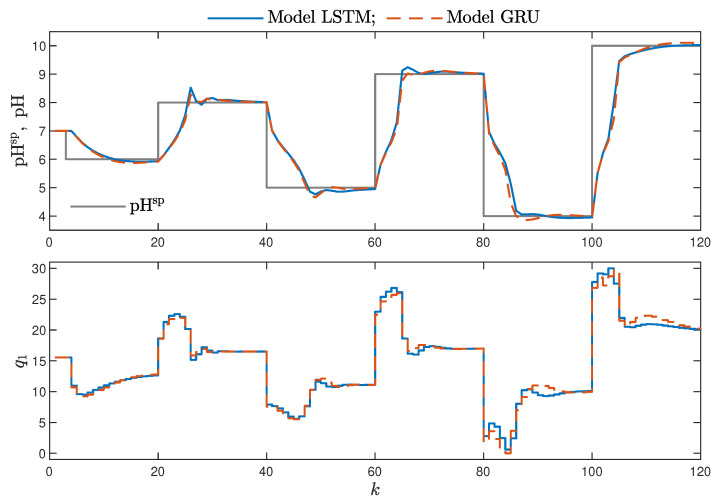
The neutralisation reactor: MPC results with the LSTM and GRU models for $n_{N}=5$, $n_{A}=n_{B}=1$.

**Figure 17:**
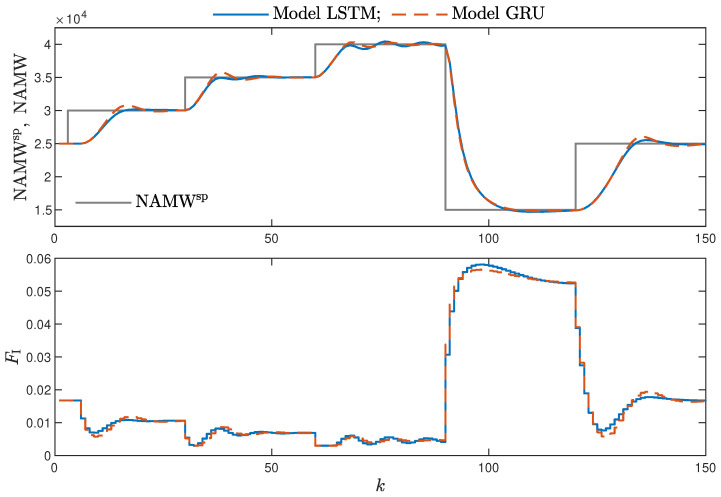
The polymerisation reactor: MPC results with the LSTM and GRU models for $n_{N}=10$, $n_{A}=n_{B}=1$.

MPC performance using the models without the recursive input signals ($n_{A}$) proved to be very satisfactory. In the case of the polymerisation reactor, in Figure 14, minimal overshoot and a short settling time can be observed. Similar control quality was achieved for the neutralisation reactor system as depicted in Figure 15. Interestingly enough, for MPC with more complex models ($n_{A}=n_{B}$), the results were comparable, as demonstrated in Figure 16. In the case of the polymerisation system and the LSTM model, small oscillations around the set-point could be observed, as shown in Figure 17, and the overall control quality was slightly worse. Table 5 and Table 6 compare the simulation results of the MPC algorithms based on the LSTM and GRU models, for the polymerisation and neutralisation processes, respectively. The following indicators used in process control performance assessment were considered [49]:

- The sum of squared errors (*E*);
- The Huber standard deviation ($\sigma^{H}$) of the control error;
- The rational entropy ($S^{r}$) of the control error.

Additionally, the average time of calculation (*t*) during the whole simulation horizon (in seconds) was specified.

**Figure 18:**
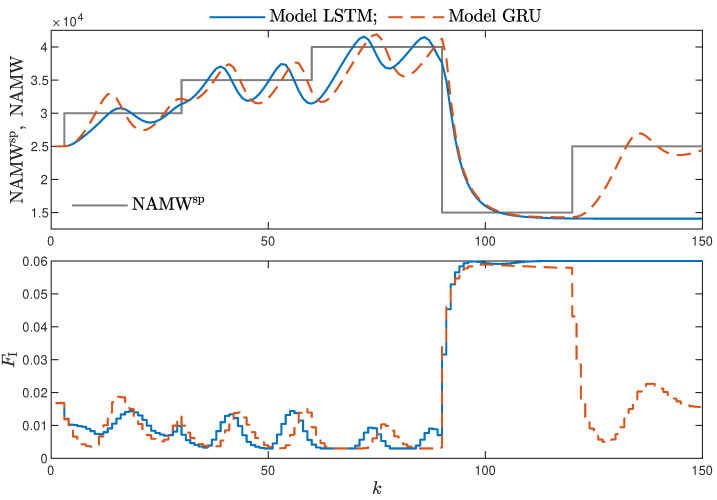
The polymerisation reactor: MPC results with the LSTM and GRU models for $n_{N}=1$, $n_{A}=n_{B}=1$.

**Figure 19:**
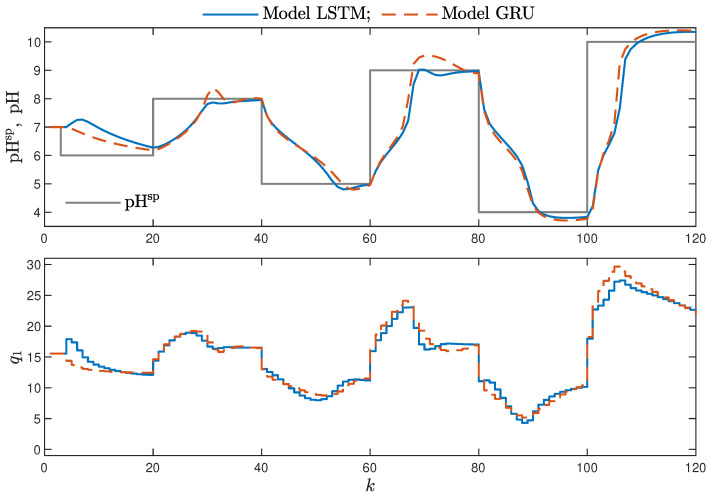
The neutralisation reactor: MPC results with the LSTM and GRU models for $n_{N}=1$, $n_{A}=n_{B}=1$.

From the performed experiments, we were able to draw the following conclusions:

1. Both types of neural networks allowed for a successful application of the MPC control scheme. All control performance indicators, i.e., *E*, $\sigma^{H}$ and $S^{r}$, showed that GRU network models, when applied for prediction in MPC, lead to very similar control quality when the rudimentary LSTM networks are used. What is more, as GRU models have fewer internal parameters, their computation cost and, therefore, the time of calculations are lower, as shown in Table 5 and Table 6;
2. It is advisable to choose models with a relatively simple structure and a low number of parameters to implement in the MPC scheme. More complex models often provide comparable or even worse quality of control, and the computation cost rises with the number of parameters of the model;
3. Minor model imperfections are reduced with great success by feedback in MPC. An example of this phenomenon can be observed in the bottom plots in Figure 12, where the model outputs differ slightly from the validation data in some areas. However, when the models are implemented in the MPC scheme, as shown in Figure 16, the quality of control is very satisfactory. However, the negative feedback is not sufficient to ensure satisfactory control if the model itself has poor quality. Example simulation results for the polymerisation process are presented in Figure 18. As a result of a very bad model, the MPC algorithm leads to unacceptable control quality, i.e., the set-point is never achieved, and strong oscillations are observed. Example simulations results when an inaccurate model is used in MPC for the neutralisation process are presented in Figure 19. In this case, the overshoot is larger and the setting time is longer when compared with the MPC algorithm based on a good model, e.g., as shown in Figure 15.

It is important to stress that the above observations are true for the two considered processes.

## 5. Conclusions

Having performed numerous experiments with different structures of LSTM and GRU neural networks as models of dynamical systems used in the MPC of two chemical reactors, we found that the GRU network gives very good results. Firstly, it approximates the properties of the dynamical systems with good accuracy, comparable with that possible when the rudimentary LSTM model is used. Secondly, it gives very good results when used for prediction in MPC, very similar to those observed in the case of the LSTM models. It is necessary to point out that the number of model parameters is lower in the case of the GRU network. Hence, the use of the GRU network is recommended for modelling of dynamical processes and MPC.

Future work is planned to develop more computationally efficient MPC control schemes based on the GRU structure and for Multiple-Input Multiple-Output (MIMO) processes. Moreover, it is planned to develop GRU models and use them in MPC applied to the ball-on-plate laboratory process [8].

## Figures and Tables

**Table 1:** The polymerisation reactor: comparison of selected LSTM and GRU networks without the recurrent inputs ($n_{A}=0$) in terms of the training ($E_{t}$) and validation errors ($E_{v}$).

		LSTM		GRU	
$n_{B}$	$n_{N}$	$E_{t}$	$E_{v}$	$E_{t}$	$E_{v}$
1	1	10.22	12.17	6.58	11.08
	2	2.51	4.06	1.58	2.26
	3	1.85	2.98	1.00	1.87
	4	1.21	1.85	0.58	0.99
	5	0.35	0.88	0.61	1.06
	10	0.08	0.19	0.29	0.65
	15	0.02	0.08	0.16	0.30
	20	0.06	0.13	0.09	0.16
	25	0.07	0.19	0.16	0.31
2	1	5.21	7.33	9.25	14.10
	2	0.83	1.46	2.56	4.58
	3	1.41	2.67	2.02	3.00
	4	0.30	0.59	0.57	1.19
	5	0.50	1.09	0.26	0.63
	10	0.06	0.19	0.19	0.39
	15	0.14	0.26	0.24	0.50
	20	0.13	0.23	0.11	0.24
	25	0.08	0.17	0.15	0.31
3	1	7.68	11.05	2.96	5.24
	2	1.37	2.42	1.09	1.76
	3	1.45	2.49	1.01	1.82
	4	0.55	1.01	0.66	1.27
	5	0.80	1.49	0.22	0.55
	10	0.08	0.18	0.10	0.21
	15	0.07	0.24	0.24	0.65
	20	0.07	0.16	0.14	0.27
	25	0.06	0.17	0.20	0.38

**Table 2:** The polymerisation reactor: comparison of selected LSTM and GRU networks with the recurrent inputs in terms of the training ($E_{t}$) and validation errors ($E_{v}$).

			LSTM				GRU			
$n_{A}$	$n_{B}$	$n_{N}$	$E_{t}$	$E_{v}$	$E_{t}^{\mathrm{rec}}$	$E_{v}^{\mathrm{rec}}$	$E_{t}$	$E_{v}$	$E_{t}^{\mathrm{rec}}$	$E_{v}^{\mathrm{rec}}$
1	1	1	2.67	3.26	6.83	7.86	3.73	5.64	8.06	11.07
		2	1.51	2.84	2.64	4.75	1.39	2.53	3.09	5.11
		3	0.23	0.37	0.59	0.95	0.33	0.55	0.66	1.08
		4	0.25	0.54	0.37	0.84	0.40	0.83	0.97	1.89
		5	0.10	0.19	0.21	0.41	0.19	0.50	0.53	1.18
		10	0.08	0.17	0.12	0.27	0.10	0.21	0.26	0.52
		15	0.06	0.10	0.10	0.18	0.19	0.40	0.44	0.89
		20	0.06	0.12	0.09	0.18	0.03	0.07	0.09	0.19
		30	0.02	0.04	0.04	0.08	0.07	0.12	0.17	0.31
2	2	1	3.27	4.50	8.13	10.29	4.07	6.24	6.53	9.78
		2	1.81	3.19	2.88	4.90	0.53	0.94	1.29	2.10
		3	0.99	1.84	1.60	2.83	0.82	1.42	1.54	2.61
		4	0.34	0.69	0.47	1.03	0.45	0.96	1.03	2.08
		5	0.18	0.37	0.26	0.59	0.22	0.42	0.55	0.97
		10	0.09	0.18	0.13	0.27	0.33	0.68	0.83	1.61
		15	0.13	0.31	0.18	0.46	0.04	0.14	0.10	0.30
		20	0.06	0.13	0.09	0.23	0.04	0.08	0.10	0.19
		30	0.03	0.06	0.05	0.10	0.08	0.14	0.19	0.33
3	3	1	1.93	3.18	5.72	8.26	1.48	2.81	2.64	4.59
		2	1.09	2.20	1.58	3.13	0.24	0.55	0.51	1.07
		3	1.05	1.89	1.39	2.58	0.86	1.95	1.29	2.79
		4	0.70	1.18	0.89	1.73	0.15	0.39	0.30	0.80
		5	0.17	0.34	0.31	0.62	0.27	0.64	0.41	1.00
		10	0.05	0.13	0.07	0.20	0.18	0.31	0.35	0.64
		15	0.10	0.20	0.14	0.32	0.08	0.21	0.16	0.39
		20	0.08	0.21	0.11	0.31	0.09	0.24	0.19	0.48
		30	0.11	0.17	0.15	0.24	0.10	0.18	0.22	0.36

**Table 3:** The neutralisation reactor: comparison of selected LSTM and GRU networks without the recurrent inputs ($n_{A}=0$) in terms of the training ($E_{t}$) and validation errors ($E_{v}$).

		LSTM		GRU	
$n_{B}$	$n_{N}$	$E_{t}$	$E_{v}$	$E_{t}$	$E_{v}$
1	1	6.56	5.14	13.07	13.03
	2	3.95	4.18	7.93	9.22
	3	3.23	4.08	6.36	6.58
	4	4.43	4.28	4.98	5.18
	5	2.55	2.81	6.06	5.84
	10	2.33	3.10	3.45	3.87
	15	2.37	2.97	4.52	4.71
	20	2.38	2.80	4.73	4.70
	30	1.47	2.04	1.03	2.01
2	1	6.33	5.05	11.57	10.80
	2	3.99	4.16	7.32	7.52
	3	3.09	3.89	6.11	6.41
	4	2.94	3.43	6.66	6.49
	5	1.29	1.85	5.96	5.33
	10	1.48	2.14	1.48	2.27
	15	1.68	2.07	1.60	2.54
	20	1.28	1.54	1.91	2.32
	30	1.15	1.69	1.38	2.37
3	1	7.79	6.88	12.27	12.10
	2	4.07	4.51	7.31	7.40
	3	3.24	4.08	6.56	7.46
	4	3.82	4.70	4.85	5.81
	5	3.47	4.11	2.86	3.79
	10	2.63	3.46	1.16	1.77
	15	1.26	1.88	1.07	1.90
	20	1.08	1.60	1.22	1.92
	30	0.94	1.75	1.21	2.11

**Table 4:** The neutralisation reactor: comparison of selected LSTM and GRU networks with the recurrent inputs in terms of the training ($E_{t}$) and validation errors ($E_{v}$).

			LSTM				GRU			
$n_{A}$	$n_{B}$	$n_{N}$	$E_{t}$	$E_{v}$	$E_{t}^{\mathrm{rec}}$	$E_{v}^{\mathrm{rec}}$	$E_{t}$	$E_{v}$	$E_{t}^{\mathrm{rec}}$	$E_{v}^{\mathrm{rec}}$
1	1	1	2.46	2.92	5.00	5.17	2.39	3.25	5.98	7.79
		2	4.22	3.91	5.11	4.50	1.62	2.28	4.38	5.73
		3	2.22	2.74	3.89	4.44	1.58	2.31	3.80	5.30
		4	3.02	3.20	4.70	4.61	1.98	2.72	3.91	4.77
		5	2.56	2.97	3.81	3.98	0.77	1.32	1.55	2.33
		10	1.55	1.96	2.36	2.72	1.62	2.26	3.42	4.50
		15	2.19	2.76	3.64	4.05	2.19	2.76	3.64	4.05
		20	1.44	2.13	2.50	3.58	1.44	2.13	2.50	3.58
		30	1.11	1.68	2.13	2.86	1.11	1.68	2.13	2.86
2	2	1	2.19	2.72	3.80	4.50	2.36	3.25	5.51	7.25
		2	2.63	3.02	5.15	5.38	1.99	2.78	4.34	5.44
		3	2.01	2.77	3.16	3.99	1.97	2.84	3.88	5.17
		4	2.74	3.43	4.14	4.61	2.36	3.19	4.32	5.28
		5	2.60	3.15	3.21	3.53	2.22	2.93	3.64	4.68
		10	1.14	1.67	1.64	2.40	1.93	2.45	3.51	3.99
		15	1.55	2.03	2.28	2.67	1.55	2.03	2.28	2.67
		20	0.93	1.29	1.45	1.82	0.93	1.29	1.45	1.82
		30	1.30	1.68	1.85	2.19	1.30	1.68	1.85	2.19
3	3	1	2.05	2.50	3.78	4.25	2.79	3.39	6.15	6.66
		2	2.87	3.34	4.11	4.22	3.91	4.37	7.75	7.13
		3	1.99	2.70	2.82	3.56	1.76	2.40	4.14	5.31
		4	2.57	3.12	3.69	3.98	1.84	2.50	3.63	4.57
		5	2.59	2.99	3.73	3.72	2.16	2.82	3.98	4.68
		10	0.76	1.22	1.36	2.12	1.69	2.42	3.47	4.50
		15	0.78	1.22	1.15	1.65	0.78	1.22	1.15	1.65
		20	1.48	2.00	2.03	2.51	1.48	2.00	2.03	2.51
		30	1.29	1.77	1.82	2.14	1.29	1.77	1.82	2.14

**Table 5:** The polymerisation reactor: quality indexes and average time of calculation comparison.

			LSTM				GRU			
$n_{N}$	$n_{A}$	$n_{B}$	E	$\sigma^{H}$	$S^{r}$	t	E	$\sigma^{H}$	$S^{r}$	t
5	0	1	2.67 × $10^{7}$	667.70	1782.41	6.67	2.76 × $10^{7}$	661.55	1608.18	7.64
6	0	1	2.71 × $10^{7}$	677.17	1758.72	6.64	2.72 × $10^{7}$	771.12	1653.33	7.68
7	0	1	2.80 × $10^{7}$	630.39	1665.62	6.50	2.73 × $10^{7}$	518.58	1568.32	7.62
8	0	1	2.75 × $10^{7}$	627.67	1758.21	6.49	2.80 × $10^{7}$	639.27	1742.63	7.89
9	0	1	2.74 × $10^{7}$	477.86	1701.53	6.52	2.75 × $10^{7}$	588.22	1603.82	8.15
10	0	1	2.79 × $10^{7}$	454.59	1659.05	6.71	2.78 × $10^{7}$	549.56	1623.56	8.05
5	1	1	2.63 × $10^{7}$	1562.43	2092.48	6.69	2.77 × $10^{7}$	509.75	1577.64	8.01
6	1	1	2.68 × $10^{7}$	1536.20	1962.02	6.64	2.77 × $10^{7}$	898.48	1743.70	7.62
7	1	1	2.73 × $10^{7}$	367.47	1587.79	6.65	2.77 × $10^{7}$	1209.39	1964.14	7.48
8	1	1	2.70 × $10^{7}$	617.22	1729.51	6.67	2.71 × $10^{7}$	759.49	1843.60	7.49
9	1	1	2.76 × $10^{7}$	455.08	1688.71	6.72	2.75 × $10^{7}$	686.34	1734.03	7.41
10	1	1	2.73 × $10^{7}$	463.27	1688.71	6.61	2.78 × $10^{7}$	469.35	1612.84	7.86
5	2	2	2.68 × $10^{7}$	839.53	1785.23	6.79	2.78 × $10^{7}$	591.74	1706.74	7.46
6	2	2	2.77 × $10^{7}$	528.51	1705.00	6.50	2.80 × $10^{7}$	860.32	1878.82	7.98
7	2	2	2.77 × $10^{7}$	826.31	1710.11	5.61	2.72 × $10^{7}$	413.74	1569.33	7.79
5	0	2	2.72 × $10^{7}$	573.75	1726.27	7.68	2.74 × $10^{7}$	824.28	1841.88	8.56
6	0	2	2.76 × $10^{7}$	458.17	1731.28	7.66	2.74 × $10^{7}$	611.42	1713.80	8.80
7	0	2	2.75 × $10^{7}$	449.80	1676.01	7.93	2.74 × $10^{7}$	499.09	1592.73	8.52

**Table 6:** The neutralisation reactor: quality indexes and average time of calculation comparison.

			LSTM				GRU			
$n_{N}$	$n_{A}$	$n_{B}$	E	$\sigma^{H}$	$S^{r}$	t	E	$\sigma^{H}$	$S^{r}$	t
5	0	1	213.67	0.21	0.53	3.47	208.404	0.18	0.48	3.73
6	0	1	208.52	0.17	0.50	3.44	209.06	0.20	0.49	4.04
7	0	1	210.56	0.26	0.53	3.46	210.63	0.19	0.49	3.86
8	0	1	212.52	0.26	0.52	3.66	212.12	0.17	0.49	3.89
9	0	1	210.51	0.25	0.56	3.64	210.49	0.18	0.48	3.88
10	0	1	211.33	0.27	0.53	3.55	210.73	0.20	0.51	3.95
5	1	1	215.70	0.33	0.56	3.78	208.59	0.19	0.50	4.07
6	1	1	220.54	0.23	0.51	3.97	214.44	0.23	0.50	3.79
7	1	1	217.20	0.19	0.52	3.76	209.26	0.21	0.51	4.13
8	1	1	219.03	0.27	0.53	3.71	213.90	0.22	0.53	4.61
9	1	1	220.60	0.52	0.59	3.86	215.56	0.20	0.52	4.23
10	1	1	225.69	0.21	0.52	3.86	208.41	0.18	0.48	4.02
5	0	2	412.73	0.22	0.48	3.48	206.90	0.16	0.46	3.99
6	0	2	218.32	0.22	0.52	3.66	215.56	0.23	0.52	4.01
7	0	2	208.80	0.18	0.51	3.66	208.98	0.20	0.51	3.84
5	2	2	227.50	0.22	0.49	4.60	217.95	0.22	0.52	4.44
6	2	2	222.80	0.25	0.51	4.34	212.76	0.25	0.52	4.69
7	2	2	217.91	0.24	0.53	4.52	221.07	0.23	0.51	4.80

## Data Availability

Not applicable.
